# Distorted Optic Nerve Portends Neurological Complications in Infants With External Hydrocephalus

**DOI:** 10.3389/fneur.2021.596294

**Published:** 2021-02-01

**Authors:** Yonatan Serlin, Gal Ben-Arie, Svetlana Lublinsky, Hagit Flusser, Alon Friedman, Ilan Shelef

**Affiliations:** ^1^Neurology Residency Training Program, McGill University, Montreal, QC, Canada; ^2^Department of Medical Imaging, Soroka Medical Center, Faculty of Health Sciences, Ben-Gurion University of the Negev, Be'er Sheva, Israel; ^3^Departments of Physiology and Cell Biology, Brain and Cognitive Sciences, Zlotowski Center for Neuroscience, Ben-Gurion University of the Negev, Be'er Sheva, Israel; ^4^Zussman Child Development Center, Division of Pediatrics, Soroka Medical Center, Faculty of Health Sciences, Ben-Gurion University of the Negev, Be'er Sheva, Israel; ^5^Department of Medical Neuroscience, Brain Repair Center, Faculty of Medicine, Dalhousie University, Halifax, NS, Canada

**Keywords:** benign external hydrocephalus, cerebrospinal fluid, optic nerve, subarachnoid spaces, increased intracranial pressure

## Abstract

**Background:** Benign external hydrocephalus (BEH) is defined by rapid increase in head circumference in infancy, with neuroimaging evidence of enlarged cerebrospinal fluid (CSF) spaces. BEH was postulated to predispose to subdural hematoma, neurocognitive impairments, and autism. There is currently no consensus on BEH diagnostic criteria and no biomarkers to predict neurological sequalae.

**Methods:** MRI-based quantitative approach was used for measurement of potential imaging markers related to external hydrocephalus and their association with neurological outcomes. We scanned 23 infants diagnosed with BEH and 11 age-similar controls. Using anatomical measurements from a large sample of healthy infants (*n* = 150), Z-scores were calculated to classify subject's CSF spaces as enlarged (≥1.96SD of mean values) or normal.

**Results:** Subjects with abnormally enlarged CSF spaces had a significantly wider and longer ON (*p* = 0.017 and *p* = 0.020, respectively), and a significantly less tortuous ON (*p* = 0.006). ON deformity demonstrated a high diagnostic accuracy for abnormally enlarged frontal subarachnoid space (AUC = 0.826) and interhemispheric fissure (AUC = 0.833). No significant association found between enlarged CSF spaces and neurological complications (OR = 0.330, 95%CI 0.070–1.553, *p* = 0.161). However, cluster analysis identified a distinct subgroup of children (23/34, 67.6%) with enlarged CSF spaces and a wider, longer and less tortuous ON, to have an increased risk for neurological complications (RR = 7.28, 95%CI 1.07–49.40).

**Discussion:** This is the first report on the association between external hydrocephalus, ON deformity and neurological complications. Our findings challenge the current view of external hydrocephalus as a benign condition. ON deformity is a potential auxiliary marker for risk stratification in patients with enlarged CSF spaces.

## Introduction

Benign external hydrocephalus (BEH) is defined by rapid increase in head circumference in infancy, with neuroimaging evidence of enlarged subarachnoid spaces (SAS) and normal or moderately enlarged ventricles (1). A population-based study in Norway found BEH incidence to be 0.4 per 1,000 live births (2). The condition is usually self-limiting and requires only clinical observation. However, despite its seemingly benign nature, BEH has been associated with non-traumatic subdural hematoma (3) and was postulated to predispose to neurocognitive impairments and autism (4–6). There is currently no consensus on cut-off values for distinction between normal and pathological extra-axial cerebrospinal fluid (CSF) spaces (7) and no biomarkers to predict neurological sequalae. The pathophysiology of BEH is not well-defined. Impaired venous outflow (8), delayed maturation of arachnoid granulations and impaired absorption of CSF were hypothesized to account for CSF accumulation, mainly within the frontal SAS (9), and interhemispheric fissure (10). These abnormalities can potentially result in elevated intracranial pressure (ICP). Impaired CSF drainage, neuroimaging evidence of cerebral venous abnormalities, optic nerve (ON) deformity and high ICP are common in patients with idiopathic intracranial hypertension (IIH). BEH has been therefore suggested to be analogous to IIH in adults (11). The aim of this study was to implement a validated MRI-based quantitative approach, for objective measurement of potential diagnostic imaging markers related to external hydrocephalus and their association with neurological outcomes.

## Methods

### Study Population

This retrospective cross-sectional study was approved by Soroka Medical Center Institutional Review Board. We searched the term “Benign External Hydrocephalus” in all reports of MRI scans performed between 2012 and 2016 at our institution. We included patients with recorded BEH characteristics [following (1)], younger than <18 month of age, with overt macrocephaly or head circumference above the 98th percentile, no clinical evidence of elevated ICP, and no prior surgical brain intervention, history of head trauma, brain infection or malformation. To examine the diagnostic validity of our quantitative approach, age-similar infants, scanned for a variety of reasons, who had normal brain MRI, normal clinical examination and normal follow-up outcomes were included. Control infants were excluded if the indication for MRI was related to potential changes in the SAS or increased ICP.

### Clinical Data

Electronic medical records of all infants were reviewed. Clinical diagnoses included any neurological disorder classified by ICD-10 codes.

### Radiological Data

A written informed consent and MRI eligibility questionnaire were obtained from all parents or legal guardians of the subjects. All subjects were imaged on a 1.5T Philips (Achieva) MRI, using a 16-channel neurovascular coil. MR venography was used for imaging of the cerebral venous system and Meckel's cave. A PRESTO, shifted-echo acquisition was used, based on a 3D T1w-FFE (fast field echo) sequence. The repetition time/echo time (TR/TE) was 35/50 ms with a flip angle of 10°. The sensitivity encoding (SENSE) parallel imaging factor was 3.7, and the in-plane voxel size was 1.0 × 1.0 mm. The slice thickness was 2.0 with 1.0 mm negative overlap. One acquisition was acquired for a scan time of 2:34 min. The contrast agent gadoterate meglumine (Gd-DOTA, Dotarem, Guerbet, France, 0.1 mmol/kg, 0.5M, 1.5 mL/s) was administered intravenously. A T1-weighted 3D TFE (turbo field echo) sequence was used for evaluation of brain tissue, ventricle volumes, CSF spaces and ON deformity. The TR/TE was 8.3/3.9 ms, prepulse inversion time of 900 ms and flip angle 8°. The in-plane voxel size was 1.0 × 1.0 mm, with 1.0 mm slice thickness. The SENSE parallel imaging factor was 2.0 with one average for a scan time of 2:36 min. Experienced neuroradiologists interpreted all studies using identical methodology. BEH was diagnosed by the presence of unusually prominent SAS with or without some degree of ventricular dilation during the time of head growth.

### Quantification of Imaging Markers

**Figure 1:**
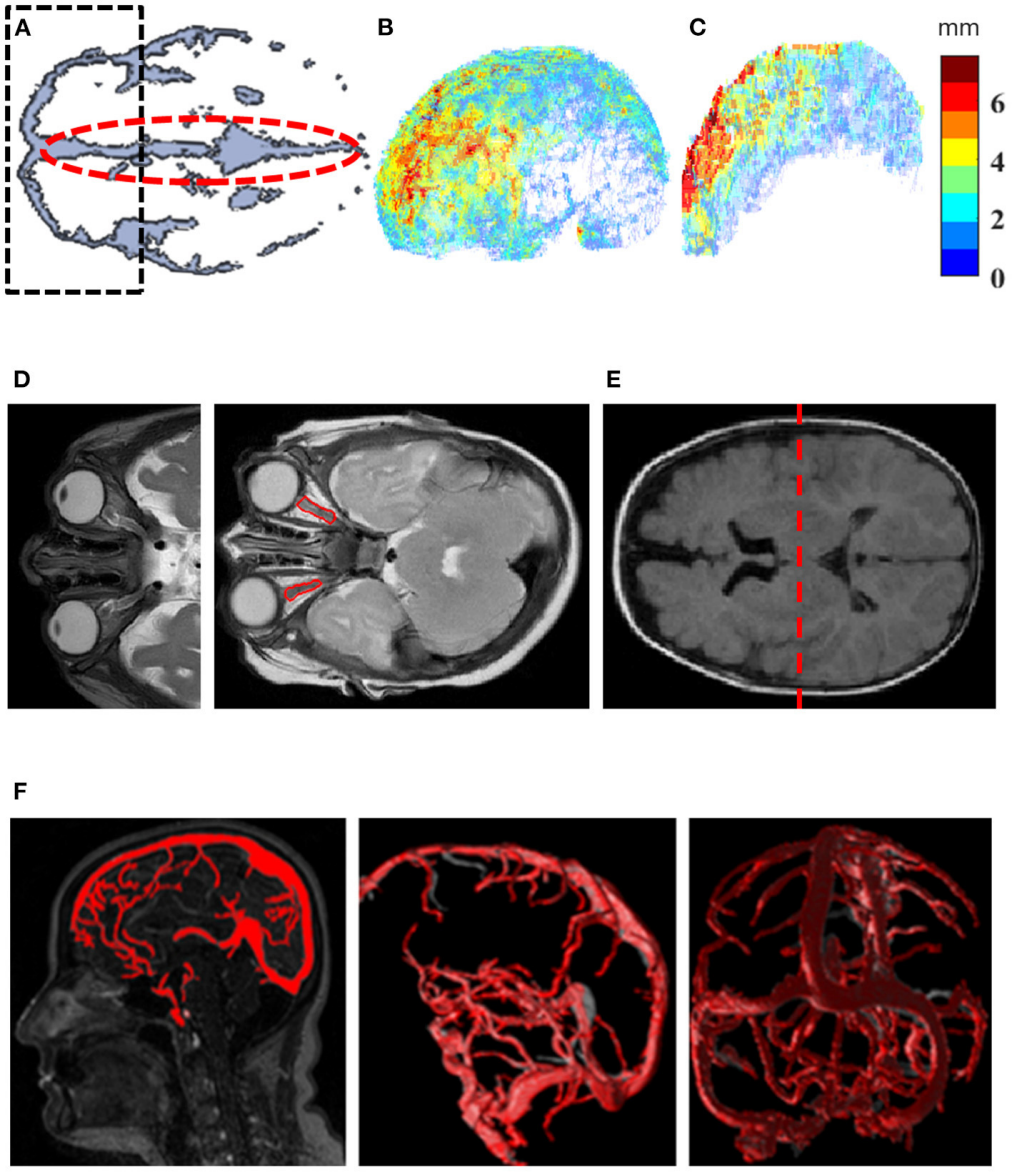
Quantification methods for investigation of imaging markers. **(A)** An axial view at the level of the Sylvian fissure was used to create a region of interest (ROI) for frontal SAS (black dashed line) and an ROI for interhemispheric fissure (red dashed line). The ROI from A was extruded along axis Z in order to create a 3D mask for the frontal SAS. **(B)** 4D visualization of SAS width map, where each voxel on the most outer surface of the SAS is characterized by its 3D position and assigned a width value (jet color-scale). **(C)** 4D visualization of interhemispheric width distribution on the mid-surface of the interhemispheric fissure. Each voxel is characterized by its 3D position and assigned a width value (jet color-scale). **(D)** Optic nerve deformity characterization (red contour). **(E)** Dividing the LV into anterior and posterior compartments (red dashed line) in the middle of the ventricular body along the Z axis to extract initial LV volume from CSF. **(F)** Automated algorithm for quantification of cross-sectional vessel alterations was used for characterization of the venous sinus system anatomy. CSF, cerebrospinal fluid; LV, Lateral ventricle; SAS, subarachnoid space.

Data were analyzed using Matlab (MathWorks Inc., USA). Figures 1A–F illustrates steps involved in the image-processing algorithm. Intracranial constituents were defined using the SPM12 toolbox (MathWorks Inc., USA). The biased-corrected T2w scans were co-registered to pre-constructed age-matched infant templates (12) and normalized to the standard Montreal Neurological Institute atlas. The normalized, biased corrected T2w were used for tissue-classification into gray matter, white matter and CSF, based on SPM12 tissue probability maps. A skull-peeled mask (total volume) was created by a combination of gray matter, white matter and CSF (Figure 1A). Estimations of frontal SAS and interhemispheric fissure widths, and assessments of ON deformity are presented in Figures 1B–D. In short, the Euclidean distance transform map was initially calculated for the CSF object. Then, 3D skeletonization of the CSF object was performed and the skeleton was subdivided into the SAS and sulcal skeletons. The SAS skeleton was defined based on its location from the outermost edge of the CSF object. The distance of 6 mm was chosen based on the reported 95th percentile value of SAS width (13) and was applied to differentiate between the SAS and sulcal skeletons. Finally, skeletons were combined with the CSF Euclidean distance transform map to estimate subarachnoid space and sulci thicknesses. A semi-automatic method was implemented for ON segmentation based on T2w as previously described (14). In short, a rough manual contour was used to define a region of interest (ROI) containing each ON. The algorithm was applied to define ON cluster within the ROI as a single 8-connected object. ON length was measured as the object skeleton length and width was derived from a combination of the ON object Euclidean distance transform map and the central line. ON tortuosity was calculated as the skeleton's arch-to-chord ratio and normalized to index values (range, 0–1, minimum to maximum). An average value from both ONs for each measurement were used. Lateral ventricle (LV) volume was assessed using the ALVIN algorithm (15) to extract initial LV volume from CSF (Figure 1E). The venous sinus system anatomy was analyzed using an automated algorithm for quantification of cross-sectional vessel alterations as previously described (16) (Figure 1F). The CSF object was adjusted by subtraction of the segmented vessel object. Segmentation of Meckel's caves by means of a region-growing algorithm applied to T2w images allowed the calculation of Meckel's cave volume (14). Neighboring voxels of manually selected seed points were iteratively examined and added to the growing object based on intensity similarity criteria.

### Statistical Analysis

Analysis was performed using SPSS 25.0 (SPSS Inc., Chicago, IL). Student's *t-*test, Mann-Whitney U, χ^2^ and Fisher's exact tests were used for comparisons. Simple and adjusted multiple linear regression models were used to evaluate the relationship between age, neuroimaging variables, frontal SAS and interhemispheric widths, and to identify new imaging markers. We excluded variables highly correlated with age or with high collinearity (*r* > 0.7). The backward variable selection method was used to detect markers significantly associated with variability in CSF spaces. To compare the extent of age-adjusted anatomical variability across subject, Z-scores were computed. Values exceeding ± 1.96 standard deviations (SD) of means (the critical Z-value for 95% confidence), were considered significantly abnormal. To evaluate the quality of the proposed imaging markers, receiver operating characteristic (ROC) analysis was used. Logistic regression applied to explore association between potential MRI markers and neurological outcomes. Odds-ratio (OR), risk-ratio (RR), and 95% confidence intervals (CI) were calculated. Two-step cluster analysis using log-likelihood distance was preformed to identify patterns of anatomical variability associated with a worse neurological outcome. Statistical significance was determined as *p* ≤ 0.05.

## Results

In total, 34 infants were included; 23 with a recorded diagnosis of BEH and 11 controls. BEH and control groups had a similar age distribution (BEH: mean, 230.2 days, range, 92–402 days; controls: mean, 246.5 days, range, 6–548 days, *p* = 0.788, *t-*test) and similar sex distribution (12/23, 52.2%, and 6/11, 54.4%, females, respectively, *p* = 0.897, χ^2^ test). There was no sex difference in patients diagnosed with BEH (*p* = 0.983, χ^2^ test). The mean percentile of head circumference was 86th for patients diagnosed with BEH and 60th for controls, who showed normal head growth over time.

### Imaging Markers Selection

Linear regression showed age to have a statistically significant association with each of the imaging variables, except for the mean width of the frontal SAS (adjusted R^2^ = 0.045, *p* = 0.119) and ON tortuosity (adjusted R^2^ = −0.030, *p* = 0.837). Measured volumes of the total intracranial compartment (combination of CSF, gray-, and white-matter), total brain tissue, lateral ventricles and cerebral venous sinuses, were highly correlated with age and/or showed high collinearity, and therefore were excluded from further analyses. ON anatomical measurements, namely width (mm), length (mm), and tortuosity index, were the only variables identified as potential independent predictors.

### ON Anatomy Is Associated With Enlarged CSF Spaces

**Figure 2:**
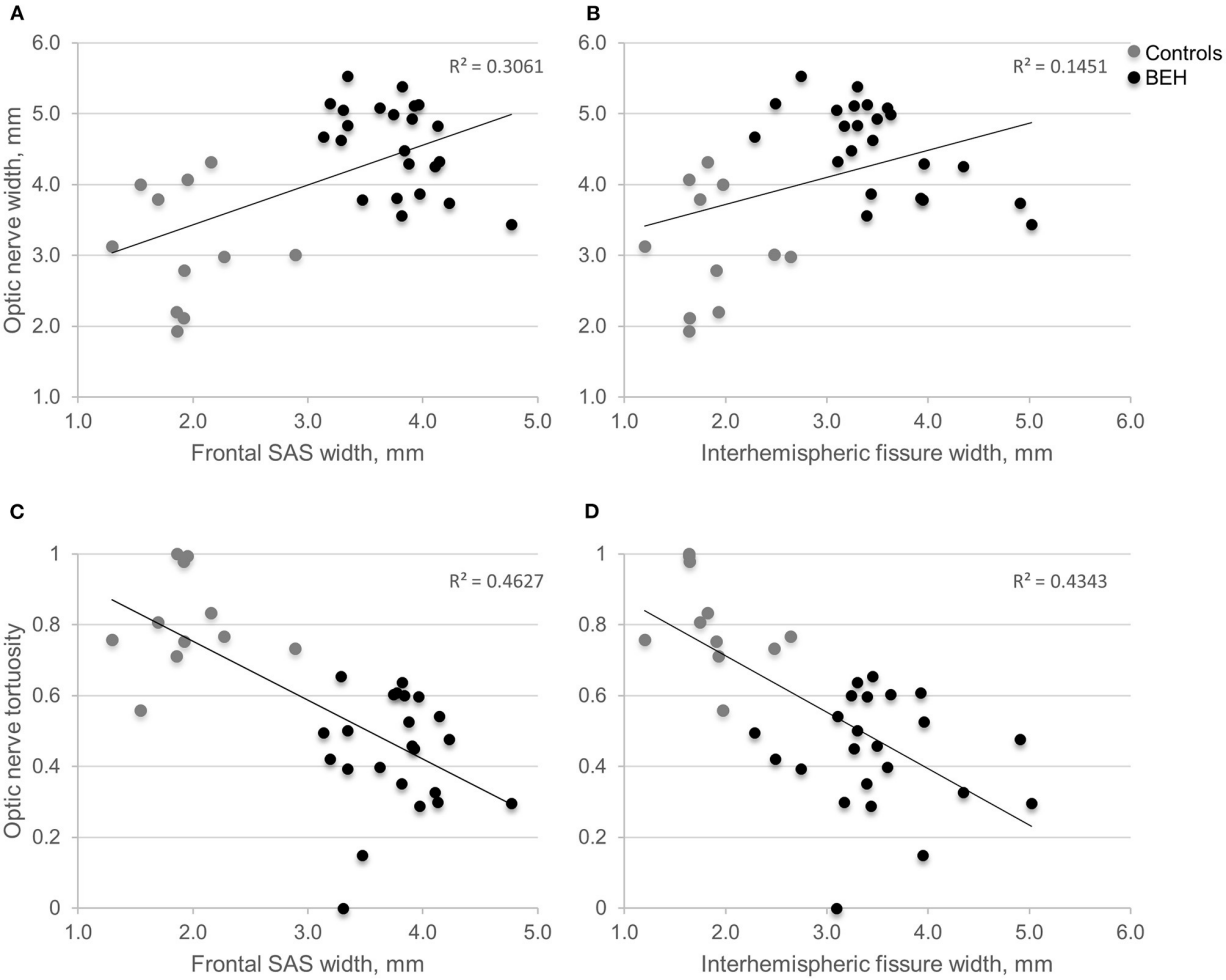
Scatter plots displaying the relationship between the extent of optic nerve width or tortuosity, and mean volume of CSF spaces. **(A,B)** As the interhemispheric fissure or frontal SAS enlarges, the optic nerve widens. **(C,D)** As the interhemispheric fissure or frontal SAS enlarges, optic nerve tortuosity declines. BEH, benign external hydrocephalus (diagnosed by neuroradiologist); CSF, cerebrospinal fluid; SAS, subarachnoid space.

Linear regression found age-adjusted ON length to have a low positive correlation with increasing frontal SAS and interhemispheric fissure widths (adjusted R^2^ = 0.182, *p* = 0.017, and adjusted R^2^ = 0.169, *p* = 0.021, respectively). Low positive correlation was also found between ON width and widening of the frontal SAS and interhemispheric fissure (adjusted R^2^ = 0.284, *p* = 0.001, and adjusted R^2^ = 0.118, *p* < 0.026, respectively, Figures 2A,B). The correlation was further improved by controlling for age (adjusted R^2^ = 0.599, *p* = 0.001, and adjusted R^2^ = 0.549, *p* < 0.001, respectively). ON tortuosity was inversely correlated with enlarged frontal SAS and interhemispheric fissure (adjusted R^2^ = 0.446, *p* < 0.001, and adjusted R^2^ = 0.417, *p* < 0.001, respectively, Figures 2C,D). The combination of quantified ON anatomical features adjusted for age, showed high association with the extent of enlarged CSF spaces. Multiple linear regression computed for prediction of patients' mean frontal SAS width, based on ON width, length, tortuosity and age, revealed a significant regression equation [F_(4, 29)_ = 22.494, *p* < 0.001], with an adjusted R^2^ = 0.723. All variables were significant predictors of enlarged frontal SAS width. Multiple linear regression model to predict mean interhemispheric width based on identical combination of variables, showed a significant regression equation [F_(3, 30)_ = 22.593, *p* < 0.001], with an adjusted R^2^ = 0.663. The final regression model, selected by means of backward elimination procedure, included ON width, tortuosity, and age as significant predictors of enlarged interhemispheric width.

### ON Anatomy as a Diagnostic Biomarker

**Figure 3:**
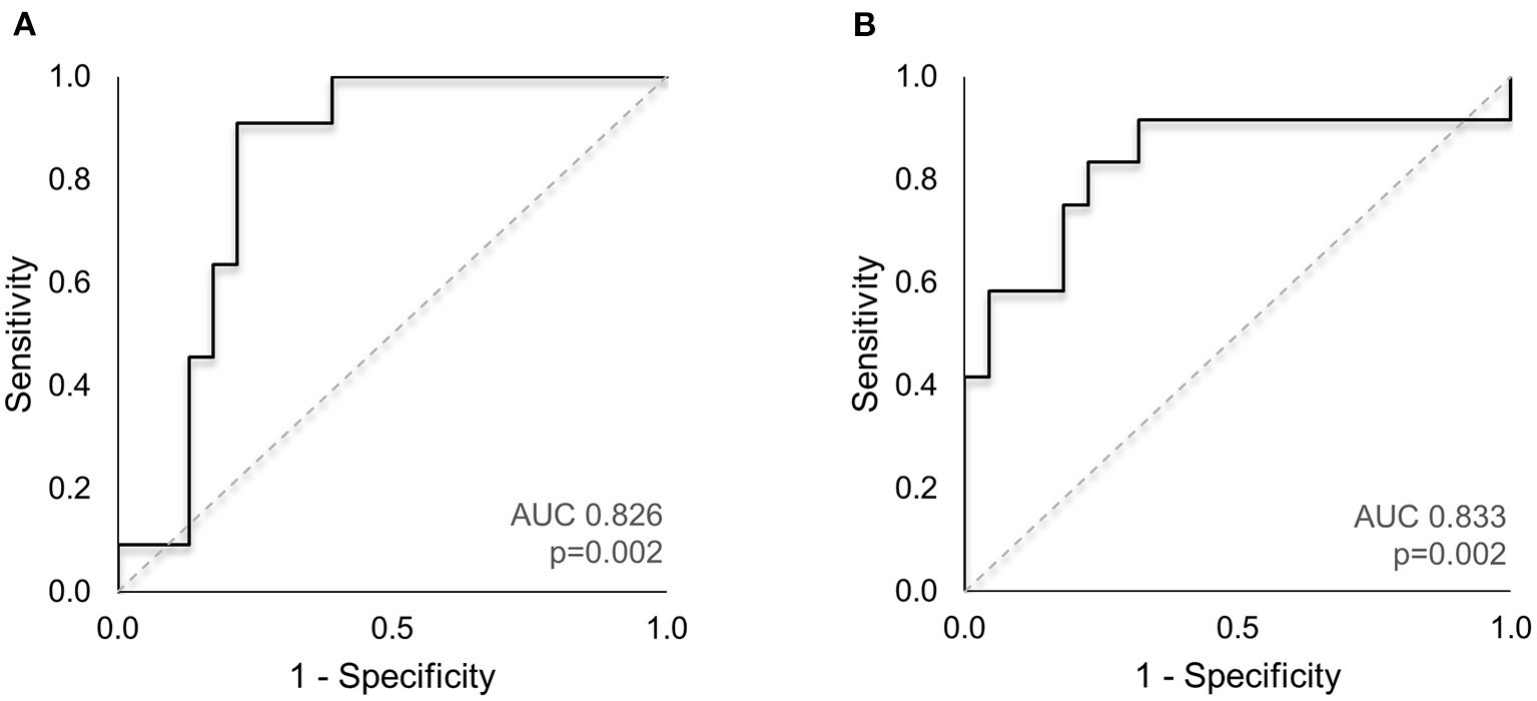
ROC analysis of ON imaging markers to identify subjects with enlarged CSF spaces. **(A)** The AUC for the optimal predictive model to identify subjects with abnormally enlarged frontal SAS (combination of ON width, length and tortuosity index) demonstrated good classification performance (AUC = 0.826, *p* = 0.002). **(B)** The best predictive model to detect subjects with abnormally enlarged interhemispheric fissure (ON width and tortuosity) showed a good diagnostic value (AUC = 0.833, *p* = 0.002). AUC, area under the curve; CSF, cerebrospinal fluid; ROC, receiver operating characteristic; SAS, subarachnoid space.

The area under the ROC curve (AUC) was used to evaluate the diagnostic relevance of ON measurements in identifying patients with enlarged CSF space. For all subjects, Z-scores of the maximal frontal SAS and interhemispheric fissure widths were computed, to determine the deviation from measurements obtained in a large sample of healthy children (*n* = 150, age <25 months) (7). We classified each subject's frontal SAS and interhemispheric fissure widths as increased (values exceeding 1.96SD) or within the normal range. ROC analysis (Figure 3) for the optimal predictive model to identify subjects with abnormally enlarged frontal SAS (ON width, length and tortuosity) demonstrated a good classification performance (AUC = 0.826, *p* = 0.002, Figure 3A). Similarly, the best model to detect subjects with abnormally enlarged interhemispheric fissure (ON width and tortuosity) showed a good diagnostic value (AUC = 0.833, *p* = 0.002, Figure 3B).

### Atypical ON Anatomy Is Prevalent in Subjects With Enlarged CSF Spaces

We next compared the magnitude of ON anatomical deviation in subjects with enlarged CSF spaces. A total abnormality score for CSF spaces ranging from 0 to 2 was calculated, based on the Z-scores of the frontal SAS and interhemispheric fissure widths. We assigned 1 point for each significantly enlarged CSF space (>1.96SD of normal). Subjects scoring 1 or 2 were considered to have abnormally enlarged CSF spaces (i.e., “quantified,” as opposed to “documented” external hydrocephalus). The 21 subjects with abnormally enlarged CSF spaces were compared to 13 subjects with CSF spaces under the 95th percentile of normal. Subjects in the “quantified” external hydrocephalus group had a significantly wider and longer ON (*p* = 0.017 and *p* = 0.020, respectively, Mann-Whitney U), and a significantly less tortuous ON (*p* = 0.006, Mann-Whitney U).

### Atypical ON Anatomy Is Associated With Increased Risk for Neurological Complications

In our cohort, long-term neurological complications (one or more witnessed seizures or documented developmental delay) were more prevalent in patients diagnosed with BEH (13/23, 56.5%) compared with controls (0/11, 0%, *p* = 0.002, Fisher's exact test). Logistic regression to determine association between increased size of CSF spaces and neurological outcomes showed no elevated risk among patients with abnormally enlarged CSF spaces (OR = 0.330, 95% CI 0.070–1.553, *p* = 0.161). When controlled for age and ON measurements, this association remained insignificant (OR = 1.162, 95% CI 0.108–12.52, *p* = 0.901). Nevertheless, a two-step cluster analysis identified a distinct subgroup of children (23/34, 67.6%), to have an increased risk for overall neurological complications (RR = 7.28, 95% CI 1.073–49.403). This group was comprised of subjects with enlarged frontal SAS (mean 3.77 vs. 1.94 mm for the “low risk” group, *p* < 0.001, Mann-Whitney U), and enlarged interhemispheric fissure (mean 3.50 vs. 1.88 mm, *p* < 0.001, Mann-Whitney U). Furthermore, comparison of the ON anatomical variability between the two subgroups revealed that subjects with an increased risk for neurological complications had a significantly wider (mean 4.56 vs. 3.12 mm, *p* < 0.001, Mann-Whitney U), longer (16.65 vs. 10.84 mm, *p* = 0.013, Mann-Whitney U), and less tortuous ON (index 0.44 vs. 0.81, *p* < 0.001, Mann-Whitney U).

## Discussion

We used a quantitative neuroimaging approach for objective detection of intracranial anatomical variability in a cohort of infants diagnosed with external hydrocephalus and controls. This is the first report on the association between external hydrocephalus, structural ON abnormalities and neurological complications. In our cohort, expanding frontal SAS and interhemispheric fissure were more likely be associated with a wider, longer and less tortuous ON. ON anatomical deviations from the norm were prevalent in subjects with enlarged CSF spaces, had a high diagnostic accuracy for external hydrocephalus and a significant predictive value for neurological complications. These associations challenge the current view of external hydrocephalus as a benign condition. Venous outflow obstruction and decreased CSF absorption have been proposed to underlie BEH and can potentially result in elevated ICP. Reports on elevated ICP in infants with BEH are lacking and measurement of CSF pressure is not part of the initial diagnostic evaluation. Open fontanelles and flexible sutures may enable the ventricles and subarachnoid spaces to expand and can explain why IIH is rarely seen in children younger than 3 years (17). The literature on ON deformity in pediatric IIH is limited to small-scale retrospective studies. Similar to our results, ON diameter was found to be significantly wider in children with IIH compared with controls (18). ON stretching due to an expanding skull has also been observed and proposed to play role in the pathogenesis of optic atrophy in children with hydrocephalus (19). Contrary to our findings, a more tortuous ON has been reported to be prevalent in children with IIH in 2 small-scale studies (20), but showed a low sensitivity of 30% and a specificity of 95% in this context (21). Tortuous ON is not an essential diagnostic criteria for IIH in children (17), and while it is unclear if BEH and IIH are indeed analogous conditions, previous pediatric studies of both entities relied on neuroradiologists to determine anatomical deformity. In the current study we used a quantitative structural assessment to accurately identify ON tortuosity, previously implemented successfully in adults with IIH (14). Rarely, isolated non-traumatic elevated ICP and increased pressure across the ON sheath can result in retinal hemorrhage (22) but there is no available data on visual impairments in children with BEH. In our institution, fundoscopy is not done routinely as part of the work up for patients with suspected BEH. Therefore, our results warrant a dedicated study to evaluate optic nerve pathology and long-term visual outcomes in patients with external hydrocephalus. In out cohort, subjects with a distinct pattern of ON anatomy appeared to have a higher risk for overall neurological complications. We hypothesize that distension of CSF spaces, in particular the peri-optic and frontal SAS, in infants with patent fontanelles, could lead to an elevated pressure gradient in a fronto-occipital direction that may result in distorted ON anatomy. Limitations of this cross-sectional, retrospective study are the unavailability of longitudinal radiological follow-ups and CSF pressure measurements, and the lack of direct fundoscopy. The relatively small control group is a general drawback of pediatric neuroimaging studies, due to ethical concerns. To increase the validity of our results we included age-similar controls and used available measurements of age-specific population means for CSF spaces. Our findings raise the potential for the use of structural MR imaging to detect ON distortion as an auxiliary marker to stratify the risk for developing visual impairments and neurological complications in patients with enlarged CSF spaces.

## Data Availability Statement

The datasets presented in this article are not readily available because protection of identifiable imaging data. Requests to access the datasets should be directed to GB-A, galben@bgu.ac.il.

## Ethics Statement

The studies involving human participants were reviewed and approved by Soroka Medical Center Institutional Review Board. Written informed consent to participate in this study was provided by the participants' legal guardian/next of kin.

## Author Contributions

YS and GB-A contributed to literature search, study design, data analysis and interpretation, and wrote the manuscript. SL contributed to data analysis and interpretation. HF, AF, and IS contributed to recruitment of participants, data acquisition, analysis, and interpretation. All authors contributed to the critical revision of the manuscript and approved the final version before submission.

## Conflict of Interest

The authors declare that the research was conducted in the absence of any commercial or financial relationships that could be construed as a potential conflict of interest.
